# A Co-Operative Perception System for Collision Avoidance Using C-V2X and Client–Server-Based Object Detection

**DOI:** 10.3390/s25175544

**Published:** 2025-09-05

**Authors:** Jungme Park, Vaibhavi Kavathekar, Shubhang Bhuduri, Mohammad Hasan Amin, Sriram Sanjeev Devaraj

**Affiliations:** College of Engineering, Kettering University, Flint, MI 48504, USA; kava6160@kettering.edu (V.K.); bhud6825@kettering.edu (S.B.); amin3672@kettering.edu (M.H.A.); deva8103@kettering.edu (S.S.D.)

**Keywords:** AD, ADAS, C-V2X, client–server protocol, GPS mapping, object detection, DNN

## Abstract

With the recent 5G communication technology deployment, Cellular Vehicle-to-Everything (C-V2X) significantly enhances road safety by enabling real-time exchange of critical traffic information among vehicles, pedestrians, infrastructure, and networks. However, further research is required to address real-time application latency and communication reliability challenges. This paper explores integrating cutting-edge C-V2X technology with environmental perception systems to enhance safety at intersections and crosswalks. We propose a multi-module architecture combining C-V2X with state-of-the-art perception technologies, GPS mapping methods, and the client–server module to develop a co-operative perception system for collision avoidance. The proposed system includes the following: (1) a hardware setup for C-V2X communication; (2) an advanced object detection module leveraging Deep Neural Networks (DNNs); (3) a client–server-based co-operative object detection framework to overcome computational limitations of edge computing devices; and (4) a module for mapping GPS coordinates of detected objects, enabling accurate and actionable GPS data for collision avoidance—even for detected objects not equipped with C-V2X devices. The proposed system was evaluated through real-time experiments at the GMMRC testing track at Kettering University. Results demonstrate that the proposed system enhances safety by broadcasting critical obstacle information with an average latency of 9.24 milliseconds, allowing for rapid situational awareness. Furthermore, the proposed system accurately provides GPS coordinates for detected obstacles, which is essential for effective collision avoidance. The technology integration in the proposed system offers high data rates, low latency, and reliable communication, which are key features that make it highly suitable for C-V2X-based applications.

## 1. Introduction

Perceiving the environment is essential to Intelligent Transportation Systems (ITS) for Autonomous Driving (AD) and Advanced Driver Assistance Systems (ADAS). Recent advancements in computer vision and Deep Neural Networks (DNNs) have achieved state-of-the-art performance in detecting objects on roads [1]. Due to recent research, applying Artificial Intelligence (AI) to computer vision, specifically Convolutional Neural Networks (CNNs), has achieved significant advancements. CNNs can learn hierarchical image features and have demonstrated exceptional effectiveness in object detection [2,3]. Nowadays, many vehicles are equipped with sensors that monitor their surroundings using AI-based environment perception algorithms. However, these environmental perception systems can generate erroneous outputs for various reasons, such as limited visibility. Although perfect environmental perception systems do not exist, many researchers are working to mitigate this issue by using redundant sensors, developing sensor-fused systems, and sharing traffic information using Vehicle-To-Everything (V2X) communication technology.

Based on the report by the National Transportation Safety Board (NTSB) [4], many studies have confirmed that V2X technology significantly improves road safety. Thus, the NTSB has consistently recommended V2X technology for its potential to prevent crashes, as demonstrated in various investigations over the years. However, there are still challenges. Many experiments for V2X capabilities are often conducted in simulation environments rather than real-world settings [5]. Connected and Autonomous Vehicles (CAVs), pivotal in advancing ITS, are the subject of significant research in both academic and industrial sectors. CAVs employ Cellular Vehicle-to-Everything (C-V2X) communication, including vehicle-to-vehicle (V2V), vehicle-to-infrastructure (V2I), vehicle-to-network (V2N), and vehicle-to-pedestrian (V2P) [6]. C-V2X communication enhances vehicle situational awareness and supports non-line-of-sight safety features for collision avoidance and accident warnings.

This paper proposes an advanced collaborative perception system for collision avoidance using C-V2X and client–server-based object detection to enhance driving safety, particularly at intersections and crosswalks. The proposed system aims to implement a low-latency, advanced co-operative environmental perception framework for collision avoidance and warning by enabling the sharing of critical traffic information essential to ADAS and AD. Unlike simulations, all test results and conclusions are based on integrating a real C-V2X communication system, ensuring practical relevance. The proposed system combines cutting-edge technologies from multiple domains, including C-V2X communication, computer vision with AI for object detection, GPS coordinates mapping from 2D image coordinates, and a client–server protocol, to enable co-operative AI.

**Figure 1:**
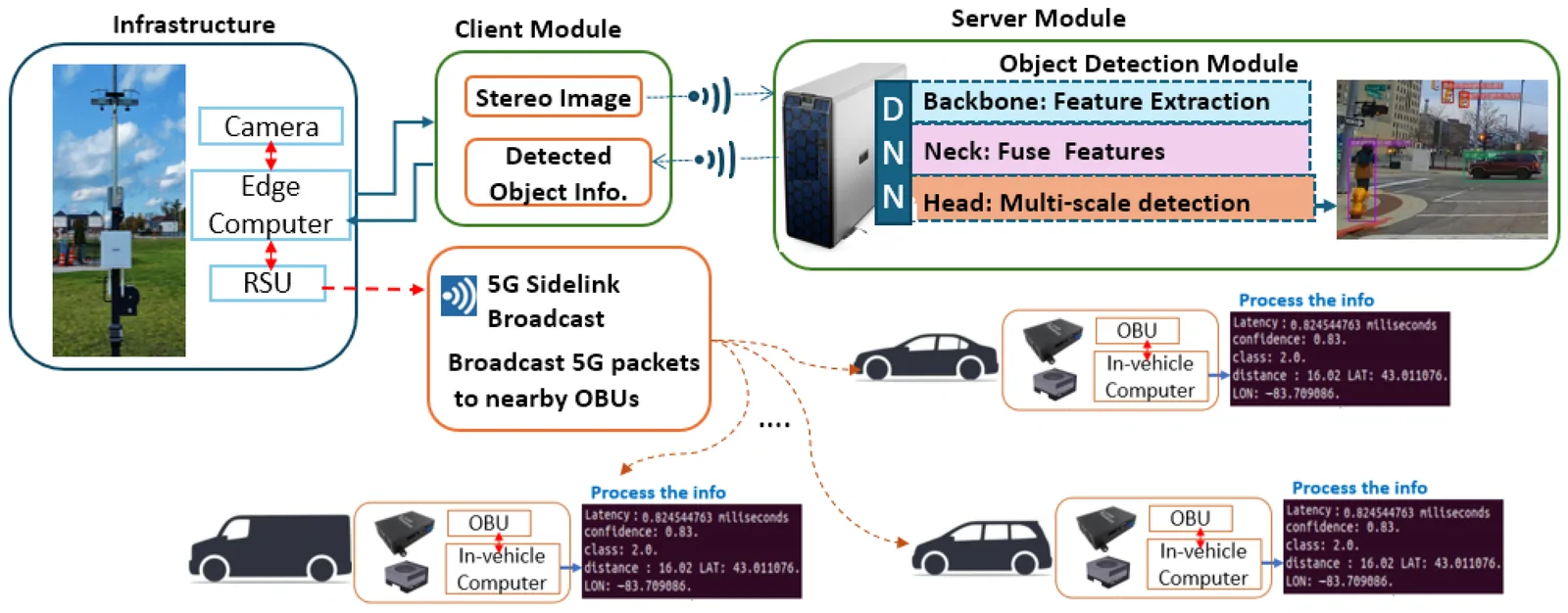
The overall architecture of the proposed co-operative perception system using C-V2X and a client–server model.

The optimal setup in C-V2X communications involves radio devices, typically called Road Side Units (RSUs) and On-Board Units (OBUs). In Figure 1, the overall architecture of the proposed system is presented. Initially, infrastructure for 5G C-V2X was deployed at the intersection area of the testing track in the GM Mobility Research Center (GMMRC) [7] at Kettering University. This infrastructure included one RSU, one camera, and one edge computer. OBUs are installed within vehicles, transmitting vehicle-specific information to the RSUs while concurrently receiving data about traffic and surrounding conditions from RSUs. The edge computing device in the infrastructure is connected to the server computer via a client–server protocol to enable rapid data processing for the DNN-based object detection algorithm. A module that maps the detected objects’ GPS locations is developed to provide helpful information about detected objects. This GPS mapping algorithm finds the corresponding GPS coordinates from the 2D image coordinates. Basic Safety Messages (BSMs) are constructed by aggregating critical traffic information, including the GPS coordinates of detected objects, their object types, and associated confidence scores. The BSMs are transmitted from the RSU to OBUs using 5G sidelink technology. This real-time traffic information sharing supports proactive collision avoidance by enabling vehicles to anticipate and respond to potential road hazards.

The remainder of this paper is organized as follows. Section 2 presents an extensive literature review on 5G C-V2X systems and their applications. Section 3 describes the methodology for developing a co-operative perception system, integrating C-V2X hardware, edge computing devices, a camera sensor paired with a DNN for object detection via a client–server protocol, and a GPS coordinate mapping module. Section 4 presents the real-time experimental results, including processing times across different computing platforms, the accuracy of GPS coordinate mapping, and C-V2X communication latency. Finally, Section 5 summarizes the key findings and insights derived from the study.

## 2. Related Work

The IEEE 802.11p [8] is an amendment of the IEEE 802.11 standard, standardizing wireless local area networks (WLANs). The 802.11p standard was specifically designed to support wireless connectivity in vehicular environments, enabling vehicles to share traffic information through direct communication with each other and with roadside infrastructure. It introduces Wireless Access in Vehicular Environments (WAVE) at both the physical and Medium Access Control (MAC) layers. The IEEE 802.11p standard [8] provides the base for the well-known early V2X technology, Dedicated Short-Range Communication (DSRC) [9]. Since its inception, DSRC has been in use for almost two decades. These technologies established the groundwork for modern vehicular communication, which is essential for developing ITSs. However, these technologies have a limited transmission range and cannot be integrated with existing cellular mobile networks. As a result, they have not achieved widespread commercial success [10,11,12].

C-V2X [13] emerged to enhance scalability, which can operate across the 5.9 GHz and cellular spectrum, thus facilitating long-range communication between vehicles and their environment. Compared to DSRC, C-V2X can cover a more extended range with higher reliability [14,15]. C-V2X, developed based on cellular systems, has evolved from Long-Term Evolution (LTE)-V2X to New Radio (NR)-V2X. It provides low-latency, high-reliability, and high-throughput communications for various C-V2X applications [11,12]. The development of the C-V2X standard within the 3rd Generation Partnership Project (3GPP) and its deployment milestones in the 5G Automotive Association (5GAA), along with the associated chipsets, were summarized in [10,11]. C-V2X can be implemented using two different transmission modes: (1) Direct Communication: Direct communication enables direct communication between vehicles, infrastructure, and road pedestrians, operating independently of cellular networks via the PC5 interface. (2) Cellular Network Communication: In this mode, C-V2X utilizes the standard mobile network to deliver information to vehicles about road and traffic conditions in the vicinity. This is achieved using the Uu interface, a radio interface that links User Equipment (UE) to the Radio Access Network (RAN) [11].

Latency remains one of the biggest challenges in deploying V2X applications. To tackle this, many researchers have explored or reviewed approaches like task offloading and resource allocation aimed at reducing latency and improving reliability in V2X systems [16,17,18,19,20,21]. In [16], the authors proposed specific resource allocation strategies and optimization methods to improve network latency performance. In the proposed methods, combining adaptive spectrum selection with hierarchical scheduling reduces latency and improves reliability. They contributed an adaptive, latency-aware approach to resource allocation. However, its real-world applicability is limited, as it depends heavily on cellular coverage and has not yet been thoroughly validated through practical testing. The authors in [17] point out that latency is one of the key challenges in rolling out LTE-V2X for AD. Their study shows that latency can be affected by several factors, such as antenna height, network coverage, and environmental conditions. On average, they observed latency around 50 ms in major European cities—but under certain conditions, it could spike to anywhere between 150 and 350 ms. In [18], a new approach, the vehicle-to-vehicle-to-infrastructure (V2V2I) paradigm for delay-aware offloading, is introduced. A source vehicle not within range of an RSU sends its data through one or more nearby vehicles using V2V communication. These intermediate hops help the data reach another car connected to an RSU, which then forwards the information via V2I. A centralized MEC server handles this by predicting which V2V2I paths can meet a set delay limit, making sure the data is delivered on time. The authors say their simulations show that this innovative, delay-aware offloading method works better than the traditional approach, where offloading only happens if the source vehicle is directly connected to an RSU. However, because their evaluation is based solely on simulations, its immediate real-world applicability may be limited. The authors in [20] introduce how V2X is being developed within 3GPP, especially as mobile networks move toward 5G with ultra-reliable, low-latency communications support. They highlight ongoing efforts by 3GPP and oneM2M to improve end-to-end data delivery, which is really important for connected vehicles. However, the paper tends to oversimplify the challenges in integrating legacy systems and edge computing, leaving important questions about operational readiness and full end-to-end reliability mostly unanswered.

In recent years, the field of AD technologies has seen the conduct of numerous scholarly surveys [22,23,24,25,26], focusing on state-of-the-art developments, conventional methodologies, advanced deep learning approaches, and strategies emphasizing communication efficiency. Tasks such as object detection and classification using DNNs are typically resource-intensive, requiring significant computational power, memory, and energy. A key challenge lies in the vast volume of sensor data processed by DNN algorithms for AD services, which can severely strain the computational efficiency of embedded systems with limited memory. This underscores the need to optimize DNN algorithms for embedded devices to improve computational performance. In [27], the authors proposed a modular edge inference framework that combines split-point selection, model compression, and task-oriented encoding to better balance computation and communication. Their approach showed latency improvements in controlled tests. However, its real-world impact remains uncertain due to the absence of validation in practical deployment scenarios. In [28], the authors suggest splitting and combining the heavy layers in DNNs to work around memory limits on edge devices. This way, the workload can be spread out across edge clusters, which reduces communication overhead and speeds up inference. Nevertheless, concerns remain regarding its scalability and robustness in real-world deployments since the evaluation was conducted in stable simulated environments.

In [29], the authors present an application of C-V2X technology for daytime visibility detection and prewarning on expressways. Their prewarning system uses C-V2X communication between the OBU and RSU, achieving a coverage radius of over 500 m and an impressively low end-to-end transmission delay of 30–35 ms. A strength of this work is its real-world implementation on an actual expressway. However, due to the absence of nearby meteorological stations and the legal and safety issues around placing distance markers on expressways, the authors were unable to calibrate the actual visibility-distance values precisely. Additionally, the detection method, which relies on contrast-based image processing, struggles with distance estimation near image boundaries, which can affect accuracy. In [30], the authors present an experimental study on a V2X application to improve road safety through a vulnerable road user collision warning system. The system works by processing camera input on the AI server to identify whether the detected object is a pedestrian or a vehicle. This information is transmitted to the RSUs via fiber optic cables and a 4G telecom network, relaying it wirelessly to vehicles in range. Each vehicle’s OBU receives the processed data and combines it with GNSS information from the car’s control unit. The study shows that the proposed system works effectively and has promising potential to improve safety in AD. However, the study also points out some limitations, namely, that manually entering geographic data such as latitude, longitude, and elevation can cause inaccuracies, potentially affecting the promptness of the warning messages.

While prior studies have made progress in vehicular communication and edge-assisted perception, they often lack real-world validation and detailed analysis of communication latency for real-time deployment. More research is needed to address this gap by implementing time-sensitive C-V2X applications and validating transmission events under realistic conditions to provide accurate latency benchmarks.

## 3. Development of a Co-Operative Perception System Using C-V2X and the Client–Server Model

To enable a co-operative perception system for collision avoidance at intersections and crosswalks, the proposed system needs the integration of several advanced technologies. These include C-V2X communication, computer vision with AI for object detection, a GPS mapping algorithm to generate objects’ GPS coordinates, and a client–server communication protocol. First, to facilitate communication between infrastructure and vehicles, deploying telematics units—specifically OBUs and RSUs—is necessary to ensure seamless data exchange. A DNN-based AI module is deployed on an edge computing device at the intersection to monitor the intersection area for real-time object detection. However, due to the high computational demands of the DNN module, a client–server architecture is used to compensate for the limited processing power of the edge computing device. The detected objects’ accurate locations must be broadcast in real time to make the camera-detected objects at the infrastructure helpful for nearby vehicles. To achieve this, this paper proposes two innovative GPS coordinate mapping algorithms to enhance the precision of object localization.

### 3.1. C-V2X Hardware Setup for Communication

**Figure 2:**
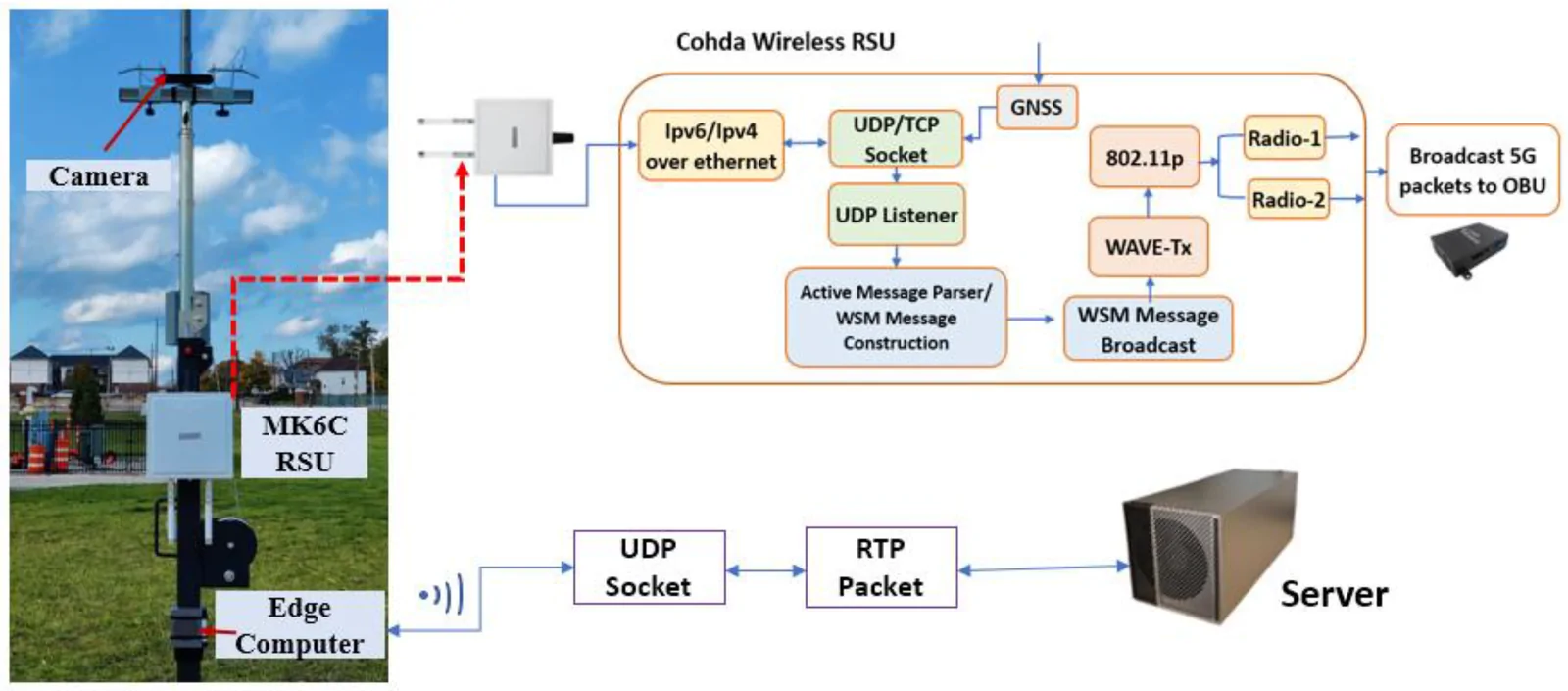
Infrastructure setup at the GMMRC testing track: one stereo camera, one computing unit, one RSU, and one server computer.

**Figure 3:**
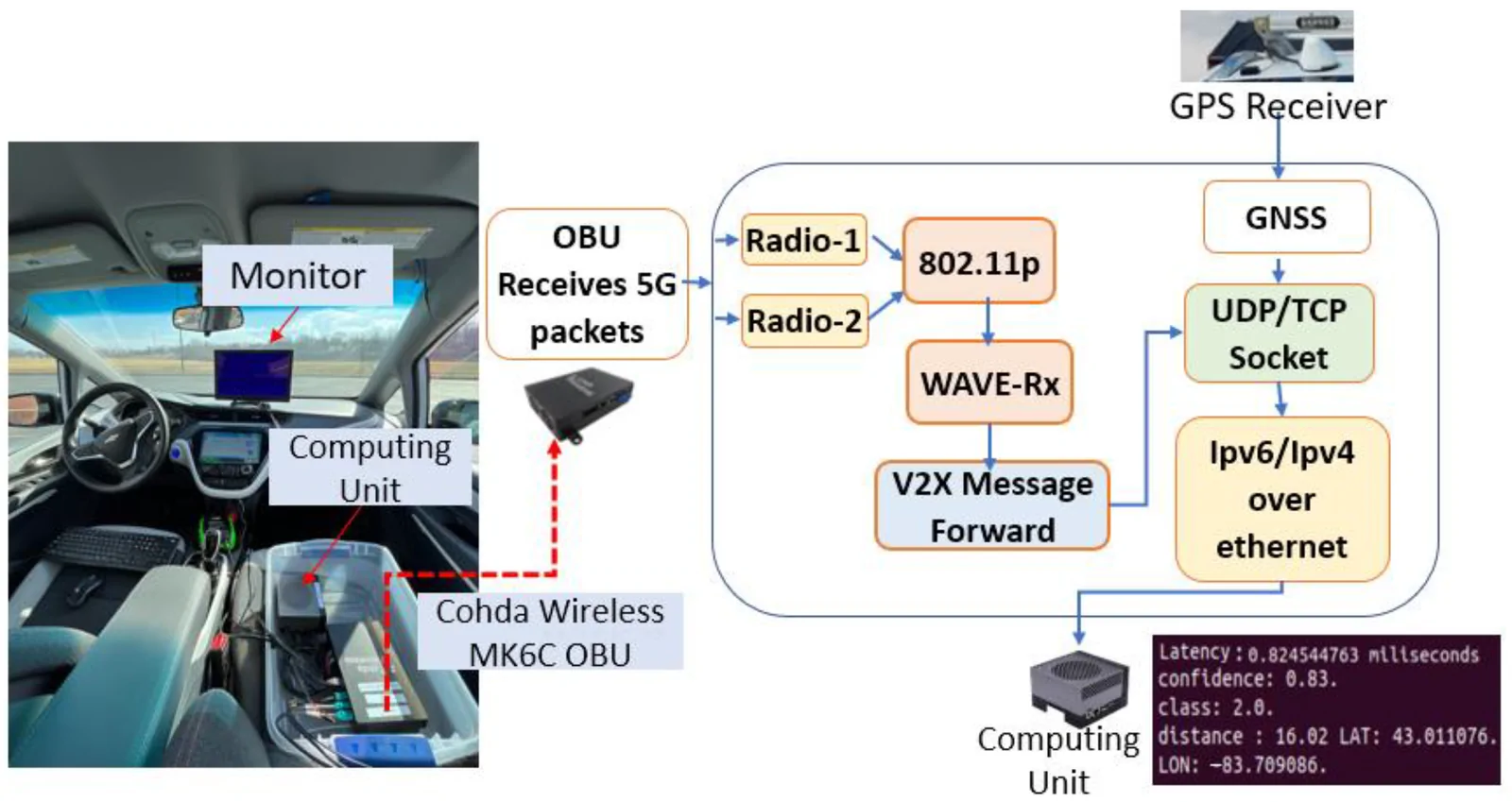
In vehicle setup for C-V2X with one OBU and one in-vehicle computing unit.

The traffic information shared through C-V2X communication improves road safety by making vehicles aware of their surroundings, such as other cars, pedestrians, and cyclists, especially near intersections. Several devices are needed to implement the C-V2X communication system for the co-operative perception system at intersection areas, including 5G communication devices, camera sensors, and edge computing devices, as shown in Figure 2 and Figure 3. As presented in Figure 2, the infrastructure consists of one RSU, one stereo camera, and one NVIDIA Jetson device. The Zed 2 camera by Stereolabs [31] was chosen to monitor the intersection area.

For the RSU device at infrastructure, the Cohda Mk6C RSU [32] was selected due to its 20 MHz bandwidth and compatibility with the 5.9 GHz ITS spectrum. Traffic information is sent from the edge computing unit to the RSU using the User Datagram Protocol (UDP), which is known for its low-latency communication. The Cohda Mk6C RSU’s communication framework, as shown in Figure 2, is specially designed for reliable communication in dynamic environments. This setup allows the RSU to broadcast WAVE short messages, which are crucial for V2X communication [33].

The OBUs can be used for smooth communication with the RSU or nearby OBUs. The OBUs are easily integrated into vehicles, as shown in Figure 3. The GPS receiver is placed on the roof of the testing vehicle. The GPS module used in this system should be placed under open-sky conditions, with the GPS signal assumed to be consistently available and unaffected by significant obstructions such as tall buildings or dense foliage. Using the Cohda Wireless MK6C EVK as the OBU [34], it provides a 20 MHz bandwidth and works within the 5.9 GHz ITS spectrum. When the OBU receives WAVE short messages, it decodes them and sends the data to the vehicle’s computing unit through an Ethernet connection with the UDP. The edge computing device processes the received information to support the vehicle’s autonomous or driver-assistance systems [33].

### 3.2. DNN Based Object Detection System

Over the last few decades, DNN-based object detection has achieved state-of-the-art performance in various computer vision tasks, even in complex environments. Among these models, YOLO (You Only Look Once) [35] has gained widespread attention and adoption due to its high inference speed and relatively strong accuracy.

The YOLO algorithm has undergone continuous updates to improve both speed and accuracy [36,37,38,39,40]. Among the many YOLO versions, YOLOv5 [38] was selected for this study because it offers a well-balanced trade-off between speed, accuracy, and ease of use—factors that align closely with our research requirements. Although newer versions such as YOLOv6 to YOLOv8 [39] provide incremental improvements, YOLOv5 remains a robust and widely adopted solution for real-time object detection tasks. Given that the proposed co-operative perception system requires low-latency processing and efficient computation on mobile platforms, YOLOv5 provides an optimal balance between detection accuracy and computational efficiency.

**Figure 4:**
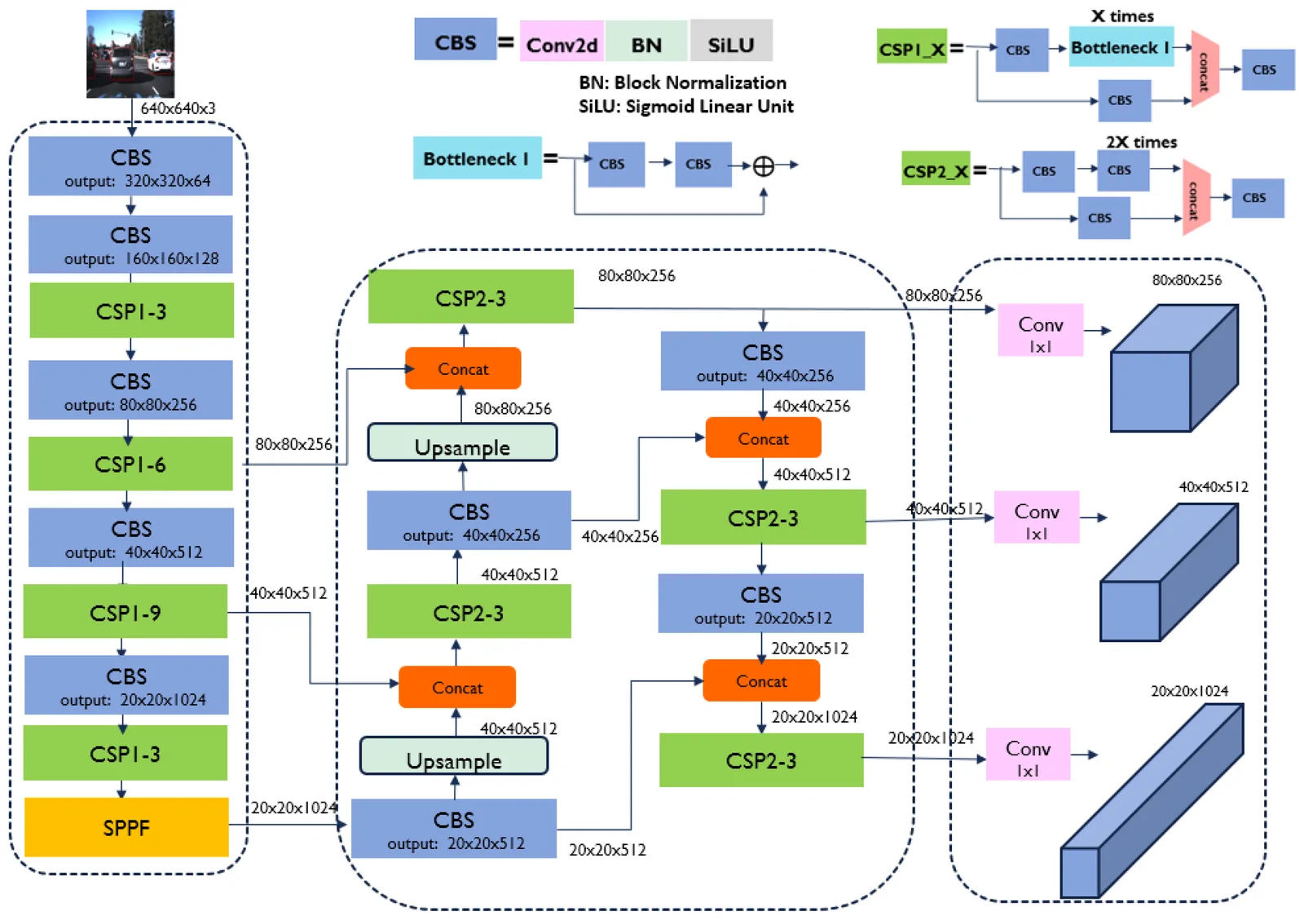
The architecture of YOLOv5.

YOLOv5 consists of the following three parts: backbone, neck, and head, as shown in Figure 4. YOLOv5 [38] uses CSPDarknet53 as a feature extraction backbone, which is inspired by CSPNet (Cross Stage Partial Network) that enhances feature learning while reducing computational cost. In the neck of YOLOv5, a combination of Path Aggregation Network (PANet) and Feature Pyramid Network (FPN) fuses information for better feature extraction [40]. PANet creates a bottom-up path to pass low-level features back into deeper layers, improving localization and small object detection. FPN is represented by the upsample and concat operations in the neck. It merges high-level semantic features from deeper layers with low-level spatial features from shallower layers, enhancing multi-scale feature detection. In YOLOv5’s head, the detection results are generated in detected objects’ bounding boxes, object detection scores, and class probabilities, similar to previous YOLO versions [40] but with improved anchor box predictions and post-processing.

**Figure 5:**
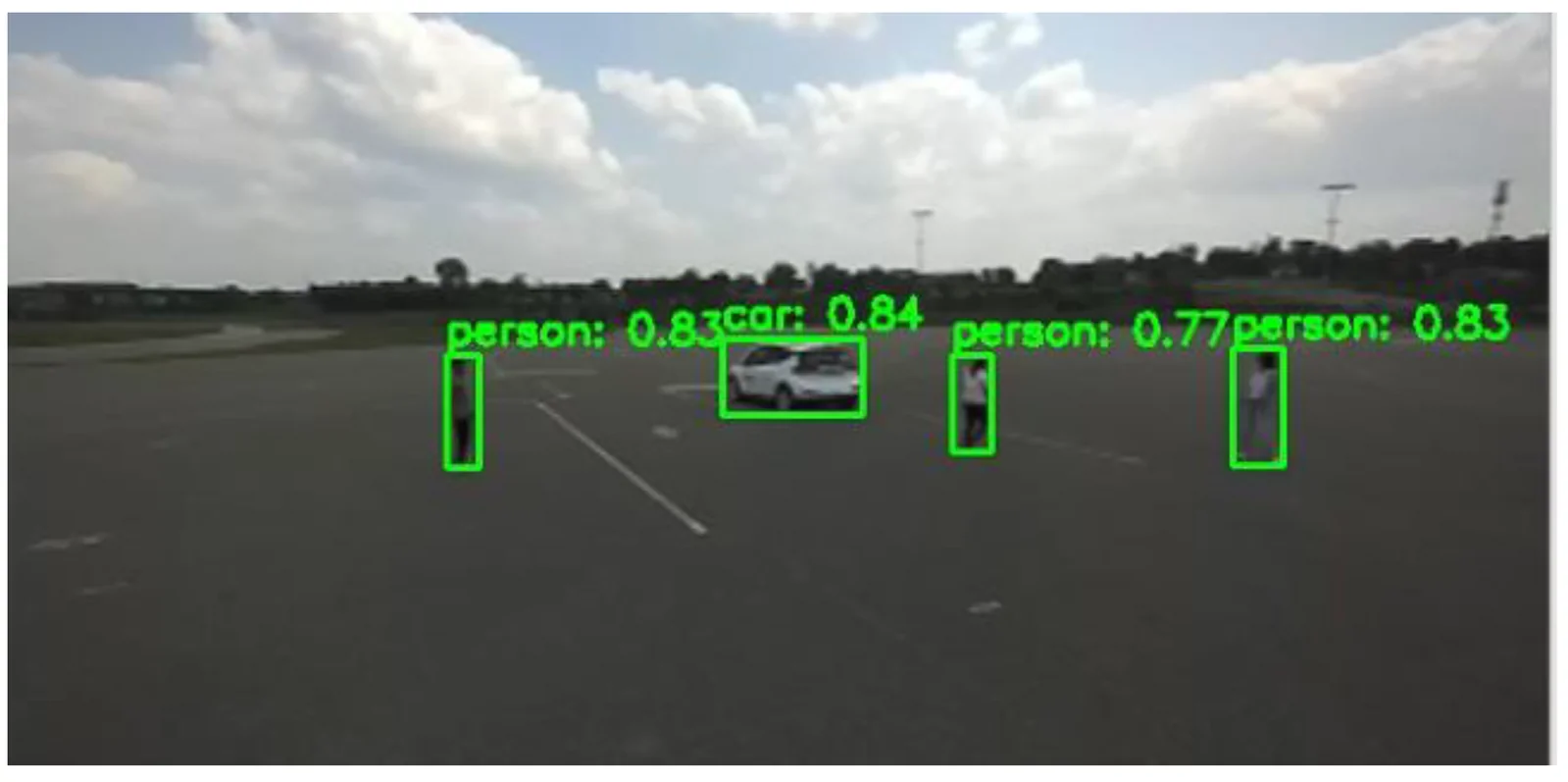
Example of object detection with YOLOv5.

Figure 5 presents the detection results by the YOLOv5 model. The system is designed to detect two types of target objects: pedestrians and cars. The demo image displays the detected object classes and their confidence scores, where the target objects are correctly classified with high confidence.

### 3.3. Client–Server Protocol for Co-Operative AI

Receiving traffic updates regarding hazards beyond the vehicle’s line of sight is critical for road safety. C-V2X applications often necessitate performing tasks that require both low latency and high computational power. A typical example is the real-time processing of image-based object recognition, such as identifying pedestrians or other vehicles, which demands extensive computation through deep CNNs [2]. In many cases, such processing demands are often beyond the capabilities of standard edge computing devices [3].

This research aims to develop a C-V2X application where the edge computing device connected to the RSU captures a livestream at an intersection, conducts object detection on the livestream, and then the RSU broadcasts to nearby OBUs by sending BSMs. However, capturing the livestream data from the camera sensor and applying the DNN-based object detection to the image data on the standalone edge computing device often increases the processing time significantly. This delay is due to hardware limitations and the high computational load of simultaneously processing the livestream and object detection on a single device. Such delays could be critical to deploying the system for real-time applications where quick decision-making is vital for road safety. In many cases, edge computing devices in the C-V2X systems have limited processing power, making it challenging to handle real-time tasks. To mitigate this issue, the client–server model is used in this research to help offload computational tasks from the edge computing device.

In a client–server model, a client is typically an application or device that requests services from a server. It generally initiates communication and is responsible for processing the information or service provided by the server. A server receives and processes client requests and then sends the necessary information or service back to the client. Servers typically have more computational power, storage, and resources than clients [41].

Two key frameworks in client–server models are the Open Systems Interconnection (OSI) Model and the Transmission Control Protocol/Internet Protocol (TCP/IP) Model, which define how computer systems communicate over a network [41]. One of the primary differences between the two models is that the OSI model divides multiple functions into separate layers, while the TCP/IP model consolidates them into fewer layers [41]. The TCP/IP model is more practical and widely used in most modern networks.

**Figure 6:**
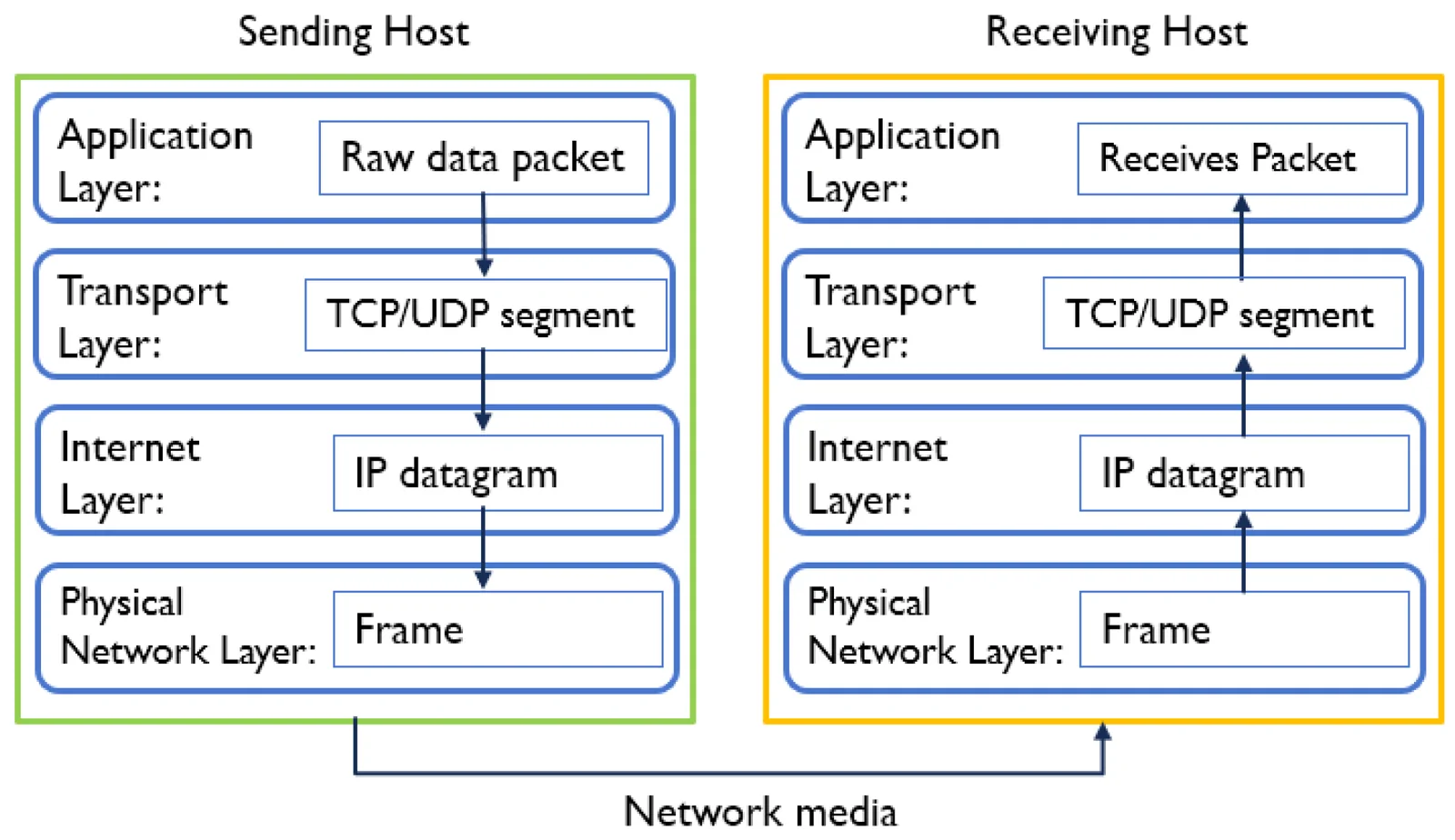
Data packet transmission between client and server in TCP/IP model.

Figure 6 presents the steps involved in the TCP/IP model. The application layer in the client generates raw data, which is passed to the transport layer and encapsulated into a TCP or UDP segment, depending on the protocol. This layer adds a header with details such as source port, destination port, and sequence number. The segment then moves to the Internet layer, where the IP protocol adds its header containing the source and destination IP addresses. Then, the IP packet is passed to the network access layer for physical transmission using Wi-Fi or Ethernet protocols [42]. On the receiving host, the process occurs in reverse order.

Socket programming establishes client–server communication, where a socket serves as an endpoint for sending or receiving data over a network. Socket programming enables data exchange between clients and servers through network connections. The client–server communication process involves capturing real-time video streams using an edge computing device attached to an RSU sending this livestream data to a server computer. The server computer conducts object detection using the DNN model, such as YOLOv5 [38]. The server identifies and locates objects in the livestream, generating bounding boxes around detected objects and classification labels (e.g., vehicle and pedestrian). After object detection and processing, the server returns the detection information to the client, which then displays the results, as shown in Figure 5.

### 3.4. Calculating GPS Locations for the Detected Objects

To develop a reliable co-operative perception system for collision avoidance, accurate locations of detected objects are critical for vehicles that utilize received traffic information. If the GPS coordinates of detected objects are broadcast, each nearby vehicle can assess potential hazards by calculating the distance between the broadcasted object’s GPS location and its own.

When a detected object is a vehicle equipped with an OBU, it can transmit its GPS information to nearby C-V2X devices. The infrastructure at the intersection—comprising a camera and an RSU—can then correlate the detected objects in the image with the received GPS data. However, this approach does not apply to all detected objects, such as pedestrians, non-equipped vehicles, or other objects without OBUs. In such cases, a mapping algorithm that estimates GPS coordinates from image pixel coordinates becomes necessary.

This paper reviews the existing mapping algorithm, homogeneous transformation, and proposes two new mapping methods to map image pixel coordinates to GPS coordinates: one based on mapping map generation and another using a machine learning-based approach.

#### 3.4.1. Mapping Through Homogeneous Transformation

The homogeneous transformation method is a mapping algorithm [29]. Equation (1) presents the homogeneous transformation method, where *M* is a projection matrix, *M* ∈ $R^{3\times 3}$.

(1) $$\left[\begin{array}{c} Lon\\ Lat\\ 1 \end{array}\right]=M\times \left[\begin{array}{c} x\\ y\\ 1 \end{array}\right]\;$$

The coordinates (*x*, *y*) in Equation (1) represent the image pixel coordinates obtained by the camera, while (Lon,Lat) denote the corresponding GPS coordinates, longitude and latitude, respectively. In Equation (1), the projection matrix *M* maps the image coordinates to GPS coordinates through a homogeneous transform [29].

If there are more than eight pairs of image pixel coordinates ($x_{i}$, $y_{i}$) and GPS points (${Lon}_{i}$, ${Lat}_{i}$), the projection matrix *M* can be found using a Direct Linear Transformation (DLT) method [29]. With the projection matrix *M*, the corresponding GPS coordinates of the given image coordinates can be found using Equation (1).

A homogeneous transformation method to map image pixels to GPS coordinates offers a simple and computationally efficient way to relate 2D image points to real-world locations, especially when the scene is approximately planar, such as flat road surfaces at intersections. It is easy to implement, works well with sufficient point correspondences, and can be made robust using techniques like RANSAC [43]. However, this method does not account for the Earth’s curvature inherent in GPS coordinates. As a result, it becomes inaccurate in non-planar scenes or larger-scale areas where 3D effects and geographic distortions are non-negligible.

#### 3.4.2. Mapping Through Map Generation

**Figure 7:**
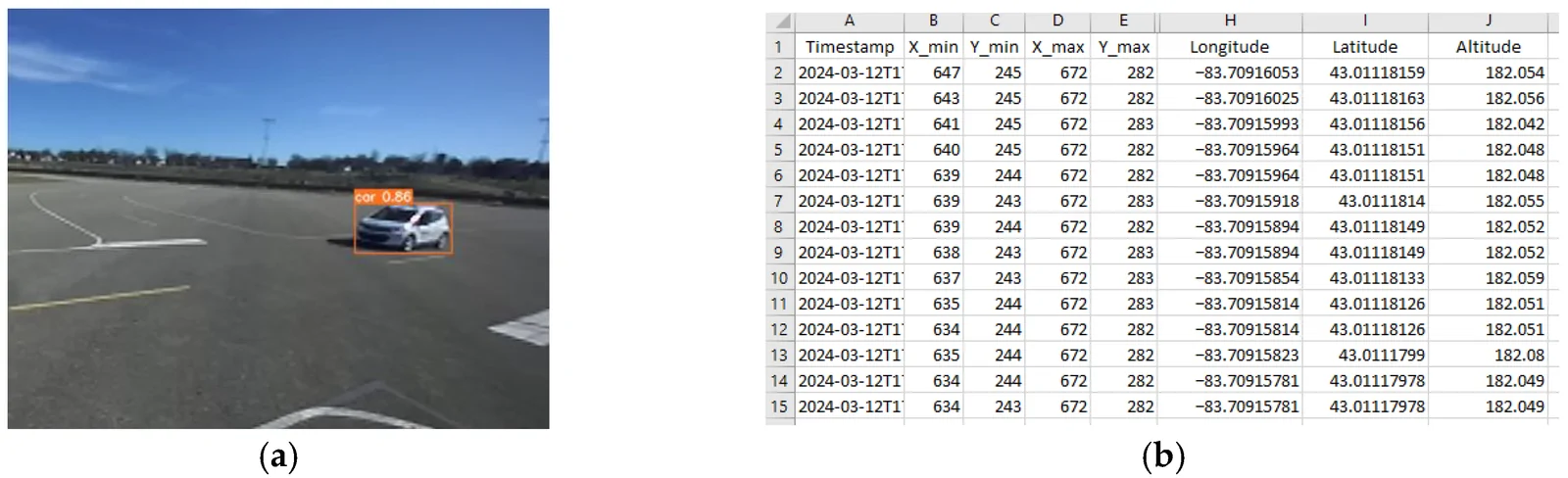
Mapping map generation: (**a**) the bounding box of the test vehicle equipped with the OBU. (**b**) The bounding box information of the detected test vehicle and the corresponding GPS coordinates from the OBU are recorded.

Mapping image coordinates to GPS coordinates can be implemented by generating a mapping map utilizing C-V2X communication. A single test vehicle equipped with an OBU drove the intersection area at the GMMRC proving ground for this task. As the car navigated the intersection area shown in Figure 7a, the OBU continuously broadcasted real-time GPS information to the nearby RSU. This information was then relayed to an edge computing device connected to the RSU. Simultaneously, the edge computer connected to the RSU used the DNN model to detect the test vehicle within camera images. The testing vehicle’s position in each frame was represented by the Bounding Box (BB) coordinates (*x*_min_, *y*_min_, *x*_max_, *y*_max_) in a 2D image, where the coordinates (*x*_min_, *y*_min_) represent the upper left corner of the BB and the coordinates (*x*_max_, *y*_max_) represent the bottom right corner of the BB. The detected vehicle coordinate in the 2D image space and the corresponding GPS coordinates from the OBU in the testing vehicle were recorded and stored together in a file, as illustrated in Figure 7b. Each data sample contains a pair of image coordinates and the corresponding GPS coordinates.

To generate the mapping map of the image coordinates to GPS coordinates, a dataset of 9031 samples was collected at the GMMRC testing track, specifically by driving through the intersection area several times. A 2D mapping map was created using collected data samples in the same size as the 2D image. The creation of the mapping map can be performed by processing the following steps:

- Set the offset pixel α=5;
- For the coordinates of the ith BB: (${x_{i}}_{min}$, ${y_{i}}_{min}$, ${x_{i}}_{max}$, ${y_{i}}_{max})$

(i) Calculate the horizontal middle coordinate of the ith BB:

(2) $${x_{i}}_{mid}=uint\left(\frac{{x_{i}}_{max}-\;{x_{i}}_{min}\;}{2}\right)$$

where uint() is a function for the unsigned integer.

(ii) Set the bottom middle coordinates of the *i*th BB as follows:

(3) $${Bottom\_i}_{mid}=\left({{{x_{i}}_{mid},\;y}_{i}}_{max}\right)$$

(iii) Using ${Bottom\_i}_{mid}$ of the ith BB, assign the corresponding GPS coordinate of the ith BB in the mapping map:

(4) $$Logitude\_map({y_{i}}_{max}-\alpha \;:{y_{i}}_{max}+\alpha ,\;{x_{i}}_{mid}-\alpha \;:{x_{i}}_{mid}+\alpha )={Lon}_{i}$$

(5) $${{Latitude\_map(y}_{i}}_{max}-\alpha \;:{y_{i}}_{max}+\alpha ,\;{x_{i}}_{mid}-\alpha \;:{x_{i}}_{mid}+\alpha )={Lat}_{i}$$

The procedure uses an offset of *α* = 5 pixels to assign identical GPS coordinates to small neighboring pixels belonging to the same object. Once the mapping maps are generated, they are used to determine the GPS coordinates for the real-time detected object in the camera image.

By utilizing C-V2X communication, mapping maps can be generated in advance, and the process becomes relatively straightforward. However, this approach can only predict the corresponding GPS coordinates for areas that were previously recorded. GPS coordinates cannot be predicted for regions that were not included in the map generation process.

#### 3.4.3. Mapping Using a Machine Learning Model

**Figure 8:**
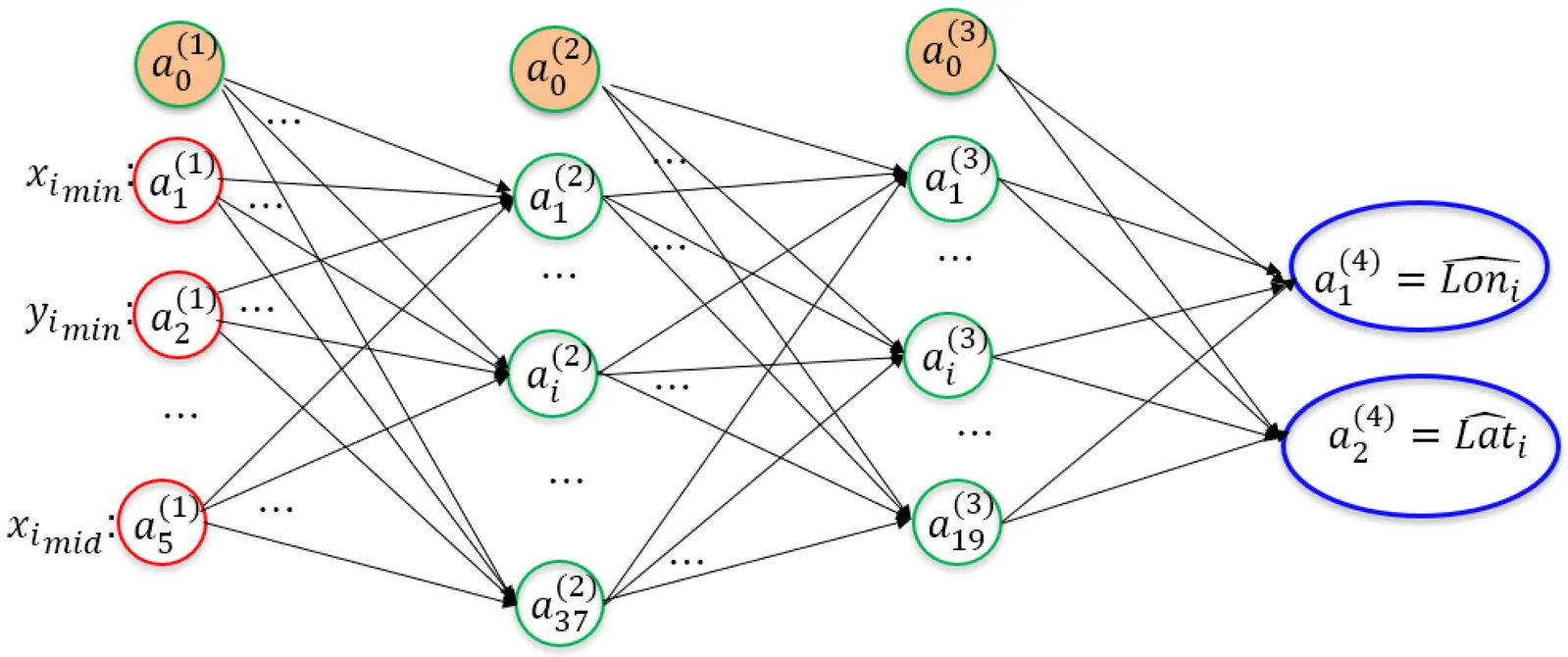
The architecture of the GPS estimation NN model.

To solve the challenge of mapping image coordinates to GPS coordinates, the dataset of 9031 samples is used to develop a Machine Learning (ML) model. Each data sample contains a pair consisting of image BB coordinates and the corresponding GPS coordinates, as shown in Figure 7b. Since the goal of the mapping is to find out the corresponding GPS coordinates given the image BB coordinates, the input to the ML model are the BB coordinates (${x_{i}}_{min}$, ${y_{i}}_{min}$, ${x_{i}}_{max}$, ${y_{i}}_{max})\;$ and the horizontal middle point of the BB, ${x_{i}}_{mid}$. The output of the ML model is the corresponding predicted longitude and latitude (${Lon}_{i},{Lat}_{i})$, as presented in Figure 8. The backpropagation Neural Network (NN) [44] was used for an ML–based mapping method that maps corresponding GPS coordinates using the bounding box information of the detected objects in 2D images.

The NNs were trained by varying the number of hidden nodes in the hidden layers, and the architecture with the best performance was selected. The Levenberg–Marquardt algorithm [45] was used to train the NNs. The Mean Squared Error (MSE) cost function shown in Equation (6) was used for the Levenberg–Marquardt optimization algorithm.

(6) $$MSE=\frac{1}{N}\sum_{i=0}^{N}\;{\left({Lon}_{i}-\hat{{Lon}_{i}}\right)}^{2}+{\left({Lat}_{i}-\hat{{Lat}_{i}}\right)}^{2}$$

where *N* is the total number of samples used during training and $\hat{{Lon}_{i}}$ and $\hat{{Lat}_{i}}$ are the NN-predicted GPS coordinates for the *i*th BB. The best-performing NN architecture with the minimal MSE was selected as shown in Figure 8.

The selected NN architecture contains four layers: one input layer with five input features, two hidden layers, and one output layer with two output nodes. The hidden layer 1 contains 37 nodes, while the hidden layer 2 contains 19. The notation $a_{i}^{j}\;$ indicates the *i*th node in the *j*th layer. The bias terms in layers were represented using the subscript 0. Among 9031 data samples, 85% of the data is used for training a backpropagation NN and the remaining 15% for validation. The NN model’s performance is measured using the Haversine formula [46]. The Haversine formula in Equations (7) and (8) calculates the distance of two GPS points on the Earth, (${{Lon}_{1},Lat}_{1})$ and (${{Lon}_{2},Lat}_{2})$, where *R* is the Earth’s radius (mean radius = 6,371,000 m).

(7) $$a={sin}^{2}\left(\frac{L{at}_{1}-{Lat}_{2}}{2}\right)+\cos\left(L{at}_{1}\right)\cdot \cos\left(L{at}_{2}\right)\cdot {sin}^{2}\left(\frac{L{on}_{1}-{Lon}_{2}}{2}\right)$$

(8) $$d=2R\cdot atan2(\sqrt{a},\;\sqrt{1-a})$$

### 3.5. A Proposed Co-Operative Perception System Using C-V2X and the Client–Server Model

**Figure 9:**
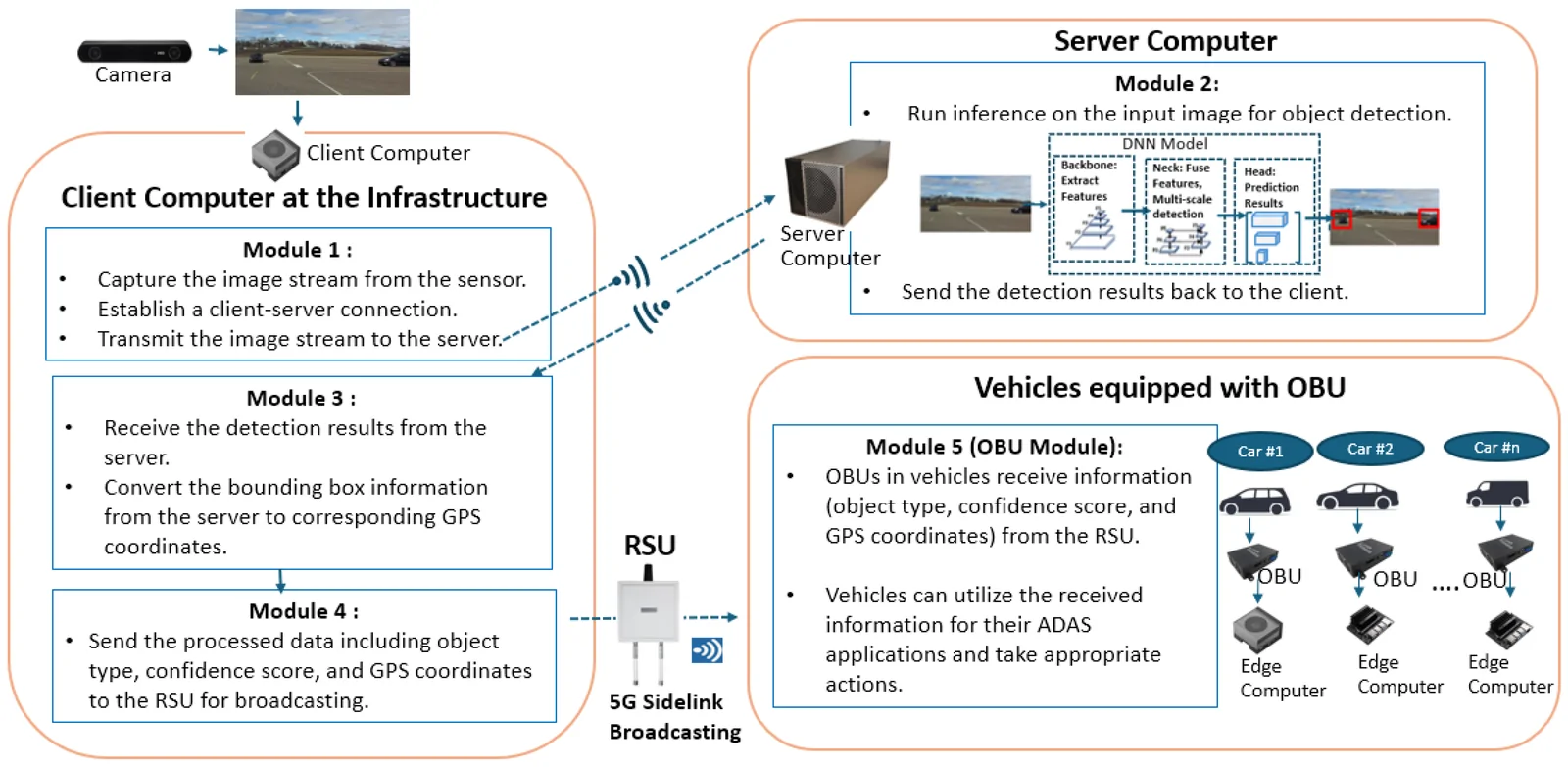
Software architecture of the proposed co-operative perception system using C-V2X and the client–server model.

The proposed system integrates recent cutting-edge technologies, including computer vision, DNNs for object detection, a client–server model, GPS mapping algorithms, and C-V2X communication to enable co-operative C-V2X for advanced perception systems at intersections and crosswalks. The system requires C-V2X hardware components, as illustrated in Figure 2 and Figure 3. Figure 9 presents the software architecture of the proposed perception system for collision avoidance using C-V2X and co-operative AI.

The steps for a co-operative C-V2X perception system for collision avoidance can be as follows: In Module 1, the system captures images of the intersection area using a camera sensor. These images are converted into byte-format BSMs by the client computer and transmitted to the server. In Module 2, the server decodes the received data and runs a DNN-based object detection algorithm, YOLOv5. The detection output—BB coordinates, object types, and confidence scores—is then encoded into byte-format BSMs and returned to the client. In Module 3, the client runs a GPS mapping algorithm to determine the GPS coordinates of each detected object, utilizing the bounding box information. In Module 4, the client transmits the processed data—including object GPS coordinates, types, and confidence scores—to the RSU using the UDP. The RSU then broadcasts the BSMs via 5G sidelink communication to nearby OBUs. In Module 5, OBUs installed in nearby vehicles forward the encoded data to their edge computing devices. These devices process the received data and can use it for their ADAS applications.

## 4. Experimental Results

The proposed co-operative perception system for collision avoidance with C-V2X and client–server protocol was fully integrated and tested at the GMMRC proving ground. The infrastructure setup—illustrated in Figure 2—was successfully completed, and four test vehicles were equipped with OBUs and edge computing devices, as depicted in Figure 3. Various testing scenarios were conducted to evaluate the following: (1) client–server model processing time, (2) accuracy of GPS mapping algorithms, (3) C-V2X communication latency, and (4) overall system deployment performance.

### 4.1. Evaluation of the Client–Server Processing Time

In this project, NVIDIA computing devices such as Jetson Nano [47] and Jetson Orin [48] are selected as edge computing devices to implement an advanced perception system using C-V2X and client–server protocol technologies. The NVIDIA Jetson devices [49] are deployed on the road, serving infrastructure and testing vehicles. These energy-efficient processing units are designed to deploy AI and robotics applications for real-time inferencing. On the other hand, the NVIDIA Drive Orin [50] device is chosen as a computing server, providing a powerful computing platform specifically designed for processing high computational tasks such as environmental perception with DNNs.

To assess the effectiveness of the client–server model in reducing processing time, five computing configurations were implemented as shown in Table 1 to run an object detection program, both with and without the client–server model. Table 1 presents the processing times, including communication overhead for configurations utilizing the client–server model if the platform uses the client–server model.

The performance comparison between the different computing platforms highlights significant differences in processing times due to the computing power of each platform. Object detection performed on the standalone Drive AGX Orin was highly effective, having an average processing time of 24.56 milliseconds (ms) due to its powerful GPU capabilities. However, the Drive AGX Orin developer kit is relatively expensive, making it unsuitable for deployment as an edge computing device on the testing track. Its high cost makes it an impractical solution for large-scale deployment, especially in testing environments where cost-effectiveness and scalability are key considerations.

The processing time difference between NVIDIA Jetson Nano and NVIDIA Jetson Orin is due to the difference in their computing hardware capabilities. The Jetson Orin has a powerful GPU and advanced architecture, including more CUDA cores, greater memory bandwidth, and a faster overall processing pipeline. On the other hand, the Jetson Nano is an affordable device with slower memory and fewer cores, resulting in higher processing time. As a result, the average processing time for object detection on the Jetson Nano is 161 ms. On the other hand, the average processing time on the Jetson Orin is 37.65 ms.

The client–server model is used to mitigate the computational power limitations of edge computing devices. Two different client–server platforms were tested. In the first setup, the Jetson Orin computer is used as the client and the Drive Orin as the server. In this configuration, the processing time is 32.83 ms. This client–server model improves the processing time by 13.26% compared to the standalone Jetson Orin model. In the second platform, the Jetson Nano is used as the client, and the Drive Orin serves as the server. This client–server model significantly reduces the processing time from 161 ms to 38.61 ms—an improvement of approximately 76%.

The client–server models can offload intensive tasks to a more powerful server (Drive Orin), and both tested configurations demonstrated significant reductions in processing time. The Jetson Orin–Drive Orin setup achieved a modest improvement of 13.26%, while the Jetson Nano–Drive Orin configuration showed a substantial 76% reduction. These results indicate that a client–server architecture can significantly enhance performance, particularly when using lower-end edge devices like the Jetson Nano, which has the lowest computing power among the tested computing devices.

### 4.2. Evaluation of C-V2X Communication Latency

Low communication latency is critical for time-sensitive applications like collision avoidance, where even delays can impact vehicle response times and overall system safety. Therefore, evaluating the communication delay within the C-V2X pipeline is essential to ensure the system meets real-time performance requirements.

To evaluate the communication delay within the C-V2X pipeline, data was transmitted from an edge computing device connected to the RSU, passed through the RSU, and then received by another edge computing device linked to the OBU. For these C-V2X latency experiments, precise time synchronization between the RSU and the OBU plays a vital role. The C-V2X communication hardware—specifically the Cohda Wireless OBU and RSU—utilizes a Network Time Protocol (NTP) daemon [51] to align system clocks with either GPS signals or NTP servers, thereby maintaining a consistent time framework. For edge computing devices, synchronization over Ethernet or Wi-Fi adjusts internal clocks to match global time standards, providing consistent timestamping.

A timestamp T_send_ was recorded just before transmission from the infrastructure-side computing device, and another timestamp T_received_ was captured upon successful reception and decoding at the OBU-side computing device. The transmission latency T_latency_ was determined using Equation (9):

T_latency_ = T_received_ − T_send_ (9)

Equation (9) captures the end-to-end delay over the network, explicitly excluding any processing or model inference time, to isolate the pure communication latency of the C-V2X link.

**Figure 10:**
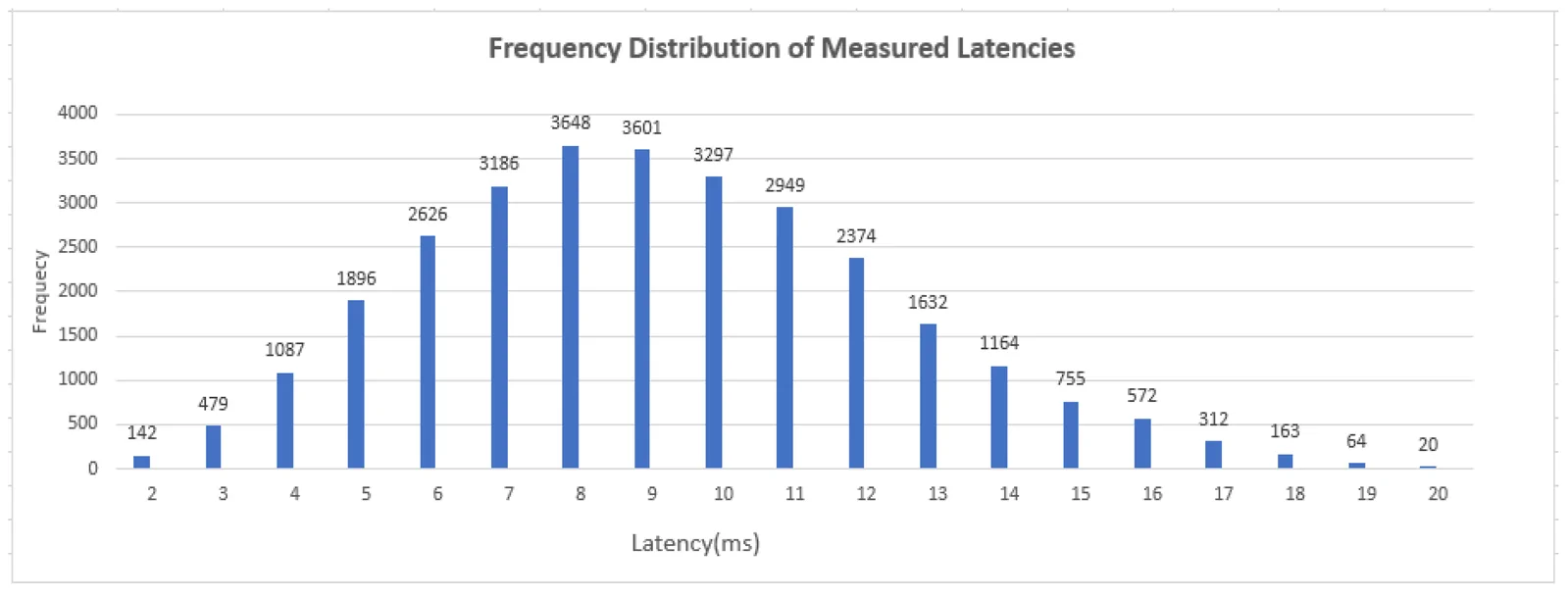
The experiment results on latency evaluation.

A total of 29,967 transmission events were evaluated, as shown in Figure 10. In Figure 10, a histogram with nineteen bins is presented, with each bin labeled on the x-axis by its center value. The histogram illustrates the frequency distribution of the measured latencies. The average latency was 9.24 ms, with a minimum latency of 2 ms and a maximum of 20 ms.

### 4.3. Evaluation of GPS Prediction Accuracy

Determining GPS coordinates from 2D image coordinates is critical for utilizing object detection results related to vehicles on the road. If the GPS coordinates of detected obstacles are broadcast by the RSU, each nearby vehicle equipped with an OBU can determine the obstacles’ locations relative to their own position.

To evaluate the accuracy of the GPS mapping algorithms, including the homogeneous transformation method [29], the proposed map-based method, and the proposed ML method discussed in Section 3.4, a test vehicle equipped with an OBU drove through the intersection and transmitted its true GPS coordinates to the RSU located at the infrastructure. The edge computer at the infrastructure performed object detection (with only one object present in the test area for this evaluation) and calculated the detected object’s GPS location. The true GPS coordinates from the OBU were then compared to the GPS coordinates produced by each mapping algorithm. Mapping accuracy was measured as the distance between the mapped and actual GPS coordinates using the Haversine formula in Equations (7) and (8). Table 2 presents the experimental results for the three mapping algorithms, obtained at the proving ground of the GMMRC. A total of 1828 testing data points were collected at the GMMRC testing track on a different day from when the training data were gathered. These points were evaluated using three different GPS mapping methods.

First, a 3 × 3 transformation matrix M was computed from the training data discussed in Section 3.4.2, using the Direct Linear Transformation (DLT) method [29] combined with RANSAC. Then, the matrix M was used to map 2D image coordinates to GPS coordinates using Equation (1). The average distance error for the homogeneous transformation method, calculated using Equations (7) and (8), was 5.1627 m with a standard deviation of 4.9129 m. The proposed map-based mapping method, which used the pre-generated map from Section 3.4.2, achieved a lower average distance error of 2.4472 m than the homogeneous transformation method. In contrast, the proposed NN-based mapping method demonstrated the best performance, with an average distance error of only 1.1914 m and a standard deviation of 2.2552 m, indicating the most consistent measurements, with values closely clustered around the average distance error.

### 4.4. Real-Time Deployment of the Co-Operative Perception System for Collision Avoidance

**Figure 11:**
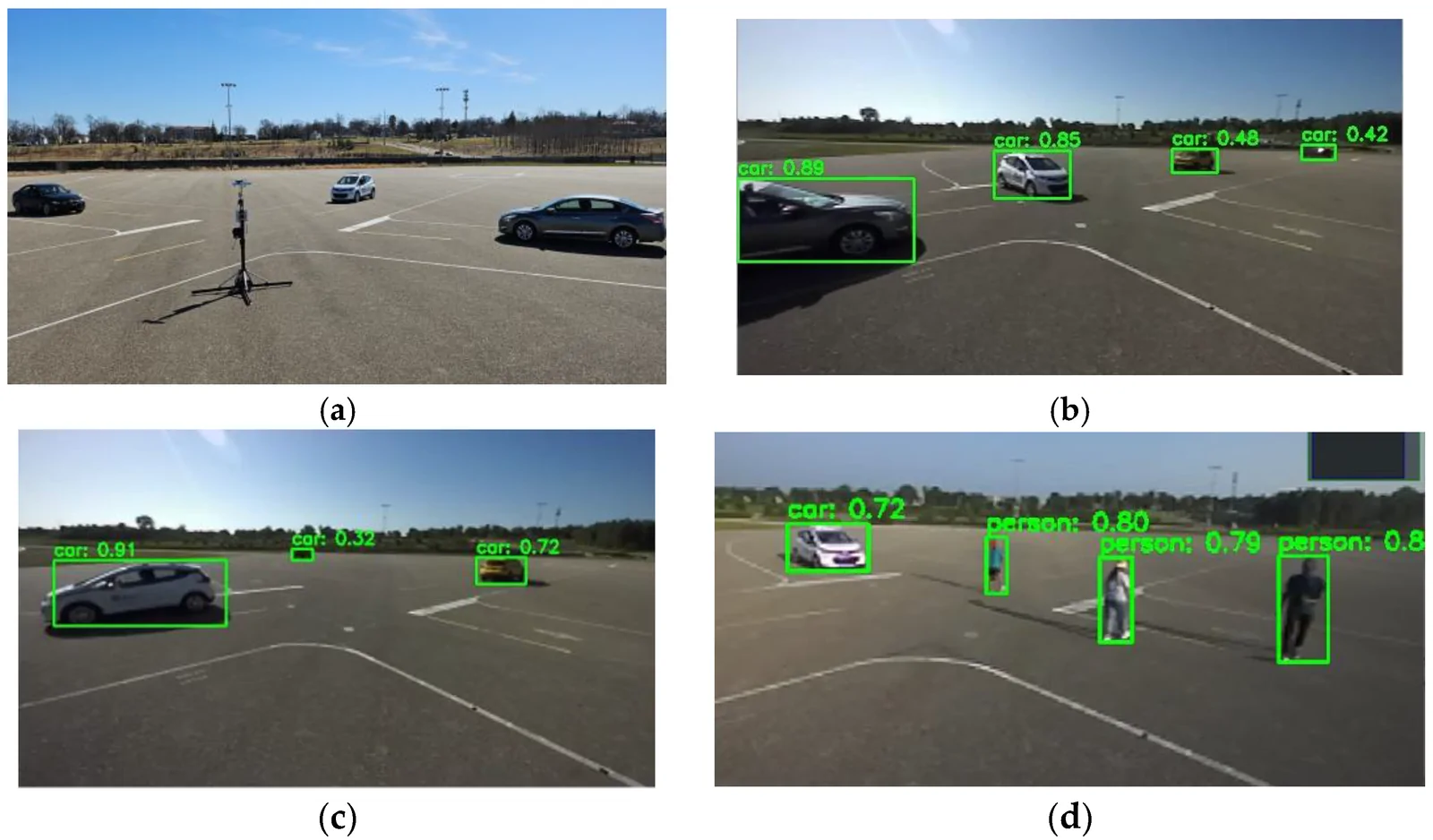
Experiments on the proving ground at GMMRC. (**a**) The GMMRC testing track. (**b**–**d**) The detected objects are represented with bounding boxes, object types, and confidence scores.

The entire system was tested using four test vehicles to evaluate the proposed co-operative perception system for collision avoidance at the GMMRC proving ground at Kettering University, as shown in Figure 11a. The evaluation system utilizes a client–server platform, with the Jetson Orin serving as the client and the Drive Orin as the server for object detection. It also employs the NN-based GPS mapping algorithm for localization.

**Figure 12:**
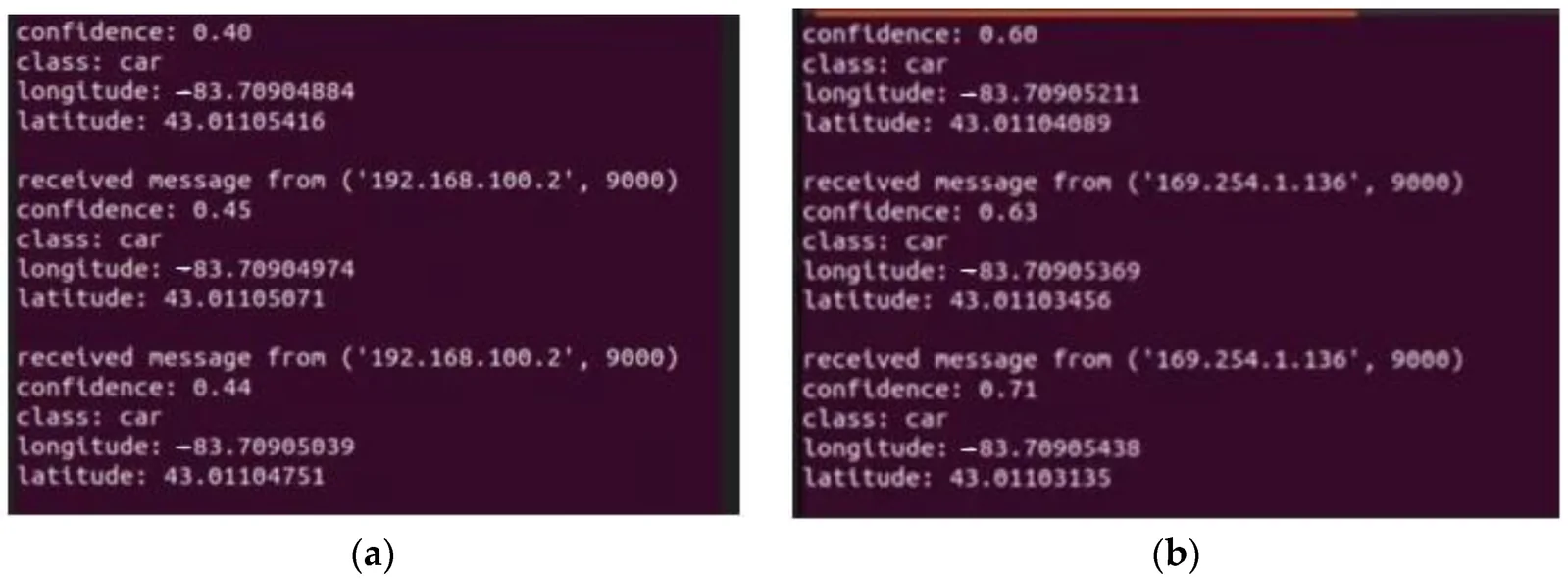
Examples of broadcast traffic information from the RSU to the vehicles’ OBUs. (**a**,**b**) Received traffic information, including object type, confidence score, and GPS coordinates.

Figure 11b–d show examples of object detection results displayed on the computer monitor at the infrastructure. In Figure 12b,c, the test vehicles are shown driving through the intersection and are successfully detected. Figure 11d illustrates pedestrians crossing the road, along with the corresponding detection results. The processing time for object detection using the client–server model—where the Jetson Orin serves as the client and the Drive Orin as the server—is 32.83 ms, corresponding to a processing rate of 30.45 frames per second (FPS). Those results demonstrate the successful implementation of the client–server model for the proposed co-operative AI.

Then, the client computer at the infrastructure maps the detected objects’ image coordinates to their corresponding GPS coordinates using the proposed ML-based mapping algorithm. The proposed GPS mapping system is implemented on an edge computer located at the infrastructure, although the system was trained using data collected from a vehicle equipped with an OBU, which was used to generate ground-truth mapping. The RSU at the infrastructure broadcasts the GPS coordinates of detected objects to nearby OBUs in vehicles. Through C-V2X communication, critical information—including the objects’ GPS coordinates, types, and confidence scores—is broadcast via PC5 sidelink from the RSU to nearby OBUs. As a result, vehicles equipped with OBUs can receive the GPS coordinates of detected obstacles from the RSU. However, the broadcasted information includes the locations of all detected obstacles, even those not equipped with C-V2X devices, such as pedestrians or vehicles without OBUs.

Figure 12 shows that test vehicles successfully received and visualized the real-time critical traffic information transmitted to their edge computing devices. This information includes object types, GPS coordinates, and confidence scores. The data is broadcast live from the RSU at the intersection to nearby OBUs in vehicles. In Figure 12a, the display monitor of one test vehicle shows the locations of three detected pedestrians. Since the GPS coordinates are provided by the RSU, the vehicle can determine the exact positions of the obstacles relative to its own location. It is important to note that these detected pedestrians do not carry any devices; rather, their positions are inferred using the mapping algorithm described in Section 3.4.3. Similarly, Figure 12b displays the GPS locations of three detected cars, which are broadcast from the RSU to the OBUs. Using the received GPS data, vehicles can accurately determine the locations of these obstacles relative to their own positions. These results show that the system combines fast object detection, accurate GPS mapping, and wide-reaching C-V2X broadcasting. The testing results prove its potential to improve co-operative safety in connected and autonomous vehicle environments.

## 5. Discussion and Conclusions

### 5.1. Discussion

The implementation and evaluation of the proposed low-latency co-operative perception system for collision avoidance reveal several key insights and implications for ITS. By integrating C-V2X communication, edge computing, and DNN-based object detection, the system presents a practical approach to enhancing road safety at intersections. Utilizing a client–server architecture effectively mitigates the limited computing capabilities of edge devices, as demonstrated by performance gains. In the first setup, a Jetson Orin is used as the client and a Drive Orin as the server. This configuration reduces processing time by 13.26% compared to the standalone Jetson Orin setup. In a more constrained scenario, the client–server model, where a Jetson Nano serves as the client and Drive Orin as the server, achieves a 76% reduction in processing time. Such improvements are critical for real-time applications, where timely detection and communication of potential hazards are essential to preventing collisions.

Additionally, the system’s average C-V2X latency of 9.24 ms highlights the technology’s suitability for time-sensitive safety applications. This low latency is vital for enabling real-time interaction between vehicles and infrastructure. The development and evaluation of two innovative GPS mapping algorithms further enhance system reliability and accuracy. The NN-based GPS mapping algorithm achieves an average localization error of 1.1914 m, outperforming the traditional homogeneous transformation-based method. Accurate obstacle localization is essential for making effective collision avoidance decisions. Field tests involving four vehicles equipped with OBUs demonstrated the feasibility of real-time object detection and information dissemination in an intersection environment. These tests validate that the integration of RSUs, OBUs, camera sensors, and edge computing can deliver scalable and practical solutions for improving intersection safety.

In addition, the proposed framework also enhances situational awareness by pinpointing detected objects on GPS and sharing that information through the RSU via the PC5 sidelink to nearby OBUs. It is worth noting that the broadcast information includes connected vehicles and unconnected road users—such as pedestrians—whose positions are inferred through the proposed GPS mapping algorithm. This feature represents a significant advancement over traditional V2X systems, which usually limit data sharing to vehicles that are already equipped. By broadcasting the GPS coordinates of detected obstacles—including vulnerable road users and other unconnected traffic participants—the system substantially broadens the scope of co-operative safety measures.

Nonetheless, certain limitations and opportunities for improvement remain. The current test scenario—conducted at the GMMRC proving ground at Kettering University—represents an idealized environment with minimal external interference. The average latency of 9.24 ms, calculated from 19,690 transmission events, provides a useful baseline under controlled conditions. However, real-world environments are more complex and subject to various sources of interference, including network congestion, environmental factors, and competing wireless devices, all of which can affect latency. Future research should focus on evaluating system performance in more dynamic and realistic scenarios to better reflect real-world deployment conditions.

Moreover, the current study does not address functional safety certification, which is crucial for real-world deployment in compliance with standards such as ISO 26262. Further research is needed to assess and ensure functional safety.

Finally, system performance may vary depending on the camera’s field of view (FOV). Incorporating multiple camera setups and leveraging sensor fusion—by combining camera data with LiDAR and radar—could significantly improve detection reliability. The proposed system was tested under clear weather conditions (e.g., no heavy rain, fog, or snow) to minimize the impact of environmental factors on camera visibility and overall system performance. Evaluation under adverse weather conditions is beyond the scope of this study and will be addressed in future work. Assessing system performance under such challenging conditions would provide valuable insights into its robustness and practical viability.

### 5.2. Conclusions

This paper presents a low-latency co-operative perception system that leverages C-V2X communication, DNN-based object detection, a client–server architecture, and GPS-based localization. The proposed system was implemented and tested in a proving ground environment, demonstrating significant latency reduction (with an average of 9.24 ms), reliable GPS-based object localization, and successful real-time C-V2X communication using OBUs and RSUs.

Several key contributions are highlighted in this work. First, scalable client–server models are implemented to offload computationally intensive tasks from resource-constrained edge devices. The performance of various client–server models is thoroughly evaluated, showcasing their impact on system efficiency. Second, two novel GPS mapping algorithms are proposed, both of which outperform traditional techniques in localization accuracy. Using the received GPS data, the vehicles can accurately determine the positions of obstacles relative to their own location. Additionally, the system’s real-time detection and communication capabilities were validated through field tests, demonstrating its potential for proactive collision avoidance.

Future work will focus on expanding the system’s field of view by integrating additional cameras, enhancing robustness through sensor fusion (e.g., by incorporating LiDAR and radar), and evaluating performance in more dynamic, real-world environments. Furthermore, addressing functional safety standards will be crucial for ensuring the system’s readiness for real-world deployment.

## Figures and Tables

**Table 1:** Comparison of processing times across different computing platforms.

Different Computing Platforms		Average Processing Time (ms)
Stand-Alone Model	NVIDIA Jetson Nano	161.00 ms
	NVIDIA Jetson Orin	37.65 ms
	NVIDIA Drive Orin	24.56 ms
Client–server Model	Client: NVIDIA Jetson Nano	38.61 ms
	Server: NVIDIA Drive Orin	
	Client: NVIDIA Jetson Orin	32.83 ms
	Server: NVIDIA Drive Orin	

**Table 2:** Performance comparison of three GPS mapping algorithms.

GPS Mapping Algorithm	Average Distance Error (m)	Standard Deviation (m)
The homogeneous transformation [29]	5.1627 m	4.9129 m
The mapping map-based method	2.4472 m	3.1522 m
The NN-based method	1.1914 m	2.2552 m

## Data Availability

The original contributions presented in this study are included in the article. Further inquiries can be directed to the corresponding author.

## Abbreviations

The following abbreviations are used in this manuscript:

AD	Autonomous Driving
ADAS	Advanced Driver Assistance Systems
AI	Artificial Intelligence
BB	Bounding Box
BSM	Basic Safety Message
CAV	Connected and Autonomous Vehicle
CNN	Convolutional Neural Network
C-V2X	Cellular Vehicle-to-Everything
DLT	Direct Linear Transformation
DNN	Deep Neural Network
DSRC	Dedicated Short-Range Communication
FOV	Field-of-View
FPS	Frames Per Second
FPN	Feature Pyramid Network
GMMRC	GM Mobility Research Center
GNSS	Global Navigation Satellite System
GPS	Global Positioning System
GPU	Graphics Processing Unit
ISO	International Organization for Standardization
ITS	Intelligent Transportation Systems
LTE-V2X	Long-Term Evolution Vehicle-to-Everything
MAC	Medium Access Control
MEC	Multi-Edge Computing
ML	Machine Learning
MSE	Mean Squared Error
NN	Neural Network
NTP	Network Time Protocol
NR-V2X	New Radio Vehicle-to-Everything
OBU	On-Board Unit
OSI	Open Systems Interconnection
PANet	Path Aggregation Network
RSU	Road Side Unit
TCP	Transmission Control Protocol
UDP	User Datagram Protocol
UE	User Equipment
UTRAN	UMTS Terrestrial Radio Access Network
V2I	Vehicle-to-Infrastructure
V2N	Vehicle-to-Network
V2P	Vehicle-to-Pedestrian
V2V	Vehicle-to-Vehicle
WAVE	Wireless Access in Vehicular Environments
YOLO	You Only Look Once
